# Development of Cross-Protective Influenza A Vaccines Based on Cellular Responses

**DOI:** 10.3389/fimmu.2015.00237

**Published:** 2015-05-15

**Authors:** Peter Christiaan Soema, Elly van Riet, Gideon Kersten, Jean-Pierre Amorij

**Affiliations:** ^1^Institute for Translational Vaccinology (Intravacc), Bilthoven, Netherlands; ^2^Division of Drug Delivery Technology, Leiden Academic Centre for Drug Research, Leiden University, Leiden, Netherlands

**Keywords:** influenza vaccines, T cell vaccines, influenza A virus, cross-reactive immune response, peptide vaccines, correlates of protection

## Abstract

Seasonal influenza vaccines provide protection against matching influenza A virus (IAV) strains mainly through the induction of neutralizing serum IgG antibodies. However, these antibodies fail to confer a protective effect against mismatched IAV. This lack of efficacy against heterologous influenza strains has spurred the vaccine development community to look for other influenza vaccine concepts, which have the ability to elicit cross-protective immune responses. One of the concepts that is currently been worked on is that of influenza vaccines inducing influenza-specific T cell responses. T cells are able to lyse infected host cells, thereby clearing the virus. More interestingly, these T cells can recognize highly conserved epitopes of internal influenza proteins, making cellular responses less vulnerable to antigenic variability. T cells are therefore cross-reactive against many influenza strains, and thus are a promising concept for future influenza vaccines. Despite their potential, there are currently no T cell-based IAV vaccines on the market. Selection of the proper antigen, appropriate vaccine formulation and evaluation of the efficacy of T cell vaccines remains challenging, both in preclinical and clinical settings. In this review, we will discuss the current developments in influenza T cell vaccines, focusing on existing protein-based and novel peptide-based vaccine formulations. Furthermore, we will discuss the feasibility of influenza T cell vaccines and their possible use in the future.

## Introduction

Several million people worldwide are infected with influenza viruses annually, which can result in hospitalization and even death from complications in severe cases. Vaccination is the preferred method to prevent influenza virus infections. Two types of influenza, influenza A and B, currently circulate among the human population. The influenza A virus (IAV), however, can be further divided in several subtypes and strains. The surface of antigens of IAV, hemagglutinin (HA), and neuraminidase (NA), frequently alter due to antigenic drift and sometimes alter due to antigenic shifts. Seasonal influenza vaccines need to be updated accordingly to match the circulating IAV strains. While seasonal influenza vaccines are effective against their matched IAV strains, they are unable to cross-react with unmatched strains. The lack of cross-reactivity of vaccine-elicited immune responses, mainly antibodies, is a major limitation of current influenza vaccines.

**Figure 1:**
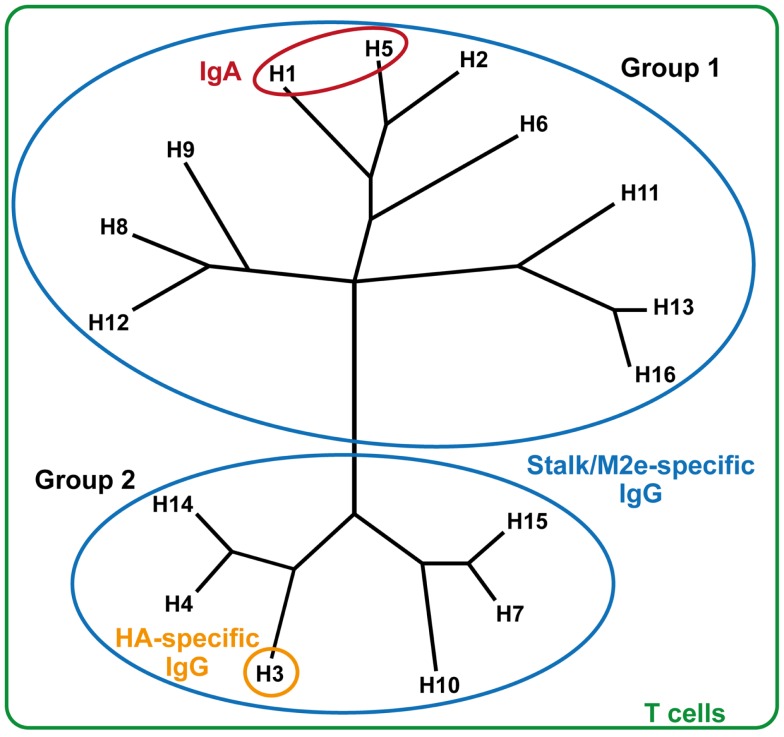
**Reactogenicity of immune responses against influenza strains**. Influenza A strains are displayed in their respective phylogenetic groups. HA-specific IgG responses (orange) react only with homologous influenza strains. Mucosal IgA responses (red) can provide heterosubtypic reactivity against related influenza strains. Stalk- or M2e-specific antibodies (blue) are cross-reactive within either group 1 or group 2 influenza strains. T cells react universally against all influenza strains, regardless of subtype or group.

Several novel concepts for the development of cross-reactive IAV vaccines have been pursued in recent years. One concept is a vaccine that induces mucosal IgA responses, which can induce strong cross-protective antibody responses against closely related IAV strains (Figure 1). However, the cross-reactivity of these IgA responses with respect to more divergent strains is modest (1). Alternatively, vaccines that induce (IgG) antibody responses against conserved antigens, such as HA stalk-reactive- or M2e-specific antibodies, might be promising (2, 3). Studies, however, indicate that these approaches mostly lead to cross-reactive responses within the same phylogenetic group of IAV, such as H5N1 and H1N1 (4), with some exceptions (5, 6). Finally, vaccines inducing influenza-specific T cell responses can offer broad and long-lasting immune responses. Since T cells recognize epitopes that are mostly derived from viral proteins located in the nucleocapsid, which are conserved between IAV strains, T cell responses can be effective against a broad range of influenza strains. This averts the necessity of seasonally changing the influenza vaccine composition, and thus could be a significant improvement over the current influenza vaccines. A drawback of a purely T cell-inducing vaccine for the prevention of seasonal influenza could be that, unlike IgA antibodies, T cell responses cannot prevent infection but prevent (severe) disease. For the application as a universal vaccine, currently T cell responses are thought to have the highest potential to induce such broad heterosubtypic responses that can react to any IAV subtype.

Natural IAV infections induce, next to antibody responses, T cell responses that are potentially cross-reactive. Indeed, it is assumed that memory T cell established by previous IAV infections prevent subsequent IAV infection in some instances; most individuals experience severe IAV-induced symptoms only a few times in their life. However, there are indications that the cytotoxic T cell (CTL) activity of T cell recall responses wanes over times in humans, suggesting that T cell responses established by IAV infections can only protect for a few years (7). Additionally, the number of available influenza-specific memory T cells should be large enough to be able to rapidly respond to IAV infection without excess additional expansion of the T cell pool (8). Furthermore, it is known that regulatory T cells suppress T cell responses during IAV infections, which can have a negative effect on the subsequent formation of a memory T cell pool (9). Natural IAV infections therefore do not mount a T cell response potent enough to provide long-lasting protection against all heterologous IAV strains. T cell-inducing influenza vaccines might overcome this shortcoming by establishing long-lasting, cross-reactive T cell responses. In this review, we will focus on the latest developments in T cell-inducing influenza vaccine research. The selection of antigen, formulation and administration strategies, as well as possible risks and limitations of T cell-inducing vaccines are evaluated.

## CD8^+^ T Cells

Primed CD8^+^ T cells, otherwise known as CTLs, are able to lyse influenza-infected cells. Via the endogenous antigen presentation pathway, infected cells will present influenza-derived epitopes on their cell surface, which are recognized by influenza-specific CTLs. The CTLs then induce apoptosis of the target cell either through the secretion of perforins and granzymes or through the Fas ligand pathway. Furthermore, CTLs produce proinflammatory cytokines such as TNF-α and IFN-γ that exert antiviral activity, which further aids viral clearance (10, 11).

Several recent studies have elucidated the importance of CD8^+^ T cells during IAV infections in humans. Sridhar et al. showed that individuals who possessed a higher frequency of CD8^+^IFN-γ^+^IL-2^−^T cells experienced a decreased clinical illness during infection with pandemic 2009 H1N1 IAV (12). CD8^+^IFN-γ^+^IL-2^−^T cells were correlated with a decreased risk of fever, an absence of viral shedding and reduced influenza-like illness (ILI) symptoms. These cells also expressed the lung-homing marker CCR5, which might explain their effectivity. CD8^+^ T cells induced by seasonal IAV strains were shown to be cross-reactive with several influenza A strains such as 2009 H1N1, swine-origin H3N2, and the recently emerged H7N9 IAV (13–15). Indeed, when cellular responses were studied in individuals infected with pandemic 2009 H1N1 IAV, rapid recall responses of CD8^+^ T cells were observed, which peaked within 1 week after infection (16). These responses were thought to originate from lymphoid memory CD8^+^ T cells established from prior seasonal IAV infections. Memory T cells were demonstrated to last for at least several years in a study, which assessed IAV-specific T cell responses in PBMCs of individuals collected from 1999 to 2012 (17). PBMCs from several donors were stimulated with Resvir-9 (a H3N2 reassortant strain), and IAV specificity and CTL activity were subsequently determined by intracellular staining with several labeled, highly conserved CTL peptides and IFN-γ.

Taken together, these studies indicate that CD8^+^ T cells can play a role in the protection against IAV infections, that these T cells are long-lived and are able to cross-react with multiple IAV strains. Thus, the induction of these T cells may be the basis of broadly reactive universal influenza vaccines.

## CD4^+^ T Cells

CD4^+^ T cells also play an important role in IAV infections, but contrary to CD8^+^ T cells, have not been studied extensively yet in humans. In animal models, activated CD4^+^ T cells can exert different roles in relation to IAV infections. CD4^+^ T cells can act as T helper cells (T_H_), providing costimulatory signals by CD40/CD40L signaling to antigen presenting cells (APCs) during the priming of B cells and CD8^+^ T cells (18, 19). Interestingly, reactivation of adoptively transferred CD4^+^ T_H_ (from IAV challenged mice) increased the recall capacity of both memory CD4^+^ and CD8^+^ T cell responses in receptive mice after IAV infection (20). While CD4^+^ T_H_ is not necessarily required for the induction of effector CTLs, it is crucial for the transition of CD8^+^ T cells to the memory phase, which is essential for the maintenance of long-lived immunity (21, 22). Surprisingly, CD4^+^ T cells can also acquire cytotoxic activity through the release of perforin in mice, providing direct protection against IAV infection (23).

In humans, it was found that pre-existing CD4^+^ T cells were reactive to pandemic 2009 H1N1 peptides, and were correlated with lower virus shedding and reduced illness during IAV infection (24). Unexpectedly, CD8^+^ T cell responses were not associated with reduced illness in this study. Nonetheless, it can be concluded that preclinical and clinical studies indicate that targeted induction of CD4^+^ T cell responses, next to CD8^+^ T cell responses, may be an attractive goal for novel vaccines.

## T Cell-Inducing Vaccines

Immune responses, and in particular, the antibodies elicited by current seasonal influenza vaccines are limited in their effectiveness against heterologous IAV infections. From the current knowledge on T cell responses during IAV infections in preclinical and clinical studies, as described above, it is believed that T cell-inducing influenza vaccines have the potential to result in broadly reactive, universal influenza A vaccines. While most vaccines are still in preclinical development, a few concepts have recently entered the clinical phase. In Table 1, the most recent developments in T cell-inducing vaccines are listed. Recently, the potency of viral vector-based influenza vaccines has been reviewed (25). In the following paragraphs, several other potential T cell-inducing influenza vaccines are highlighted.

**Table 1:** **T cell-inducing influenza vaccines in recent development**.

Class	Concept name	Antigen(s)	Adjuvant(s)	Immune response	Status	Reference
Whole virus or protein vaccine	Live attenuated influenza vaccine	Live attenuated influenza vaccine (various strains)	None	Induces CD4^+^ and CD8^+^ T cell responses in unprimed children	Licensed	(26, 27)
		Single-cycle live attenuated influenza vaccine (H3N2)	None	Induced CD8^+^ T cell responses in mice that protected against heterologous challenge	Preclinical	(28)
	Gamma-irradiated whole inactivated influenza vaccine	Whole inactivated influenza vaccine (H3N2)	None	Induces robust influenza-specific T cell responses in mice	Preclinical	(29)
	Influenza virosomes	Virosomes (H5N1)	Matrix-M	Induces good influenza-specific CD4^+^ T cell responses in healthy adults, but CD8^+^ T cell responses were limited	Phase I trials	(30)
	Multimeric-001	Synthetic protein containing B and T cell epitopes from HA, M1, and NP	Montanide ISA 51VG	Induces cellular responses in healthy adults and elderly that are reactive against multiple IAV strains	Phase I trials	(31, 32)

Peptide vaccine	Lipopeptides	Minimal T cell epitopes from M1, PA, and NS1	Pam2Cys	Induces CD8^+^ T cell responses that protect mice against heterologous IAV challenge	Preclinical	(33)
		Minimal T cell epitopes from HA and NP combined with seasonal influenza vaccine	Pam2Cys	Induces CD8^+^ T cell responses that reduces lung viral load in mice after heterologous challenge	Preclinical	(34)
		Minimal T cell epitope from NP	Phosphatidylserine	Induces peptide-specific CD4^+^ and CD8^+^ T cell responses in mice	Preclinical	(35)
	Liposome-conjugated peptides	Minimal T cell epitopes from M1, NP, PA, PB1, or PB2	Liposomes, CpG-ODN 5002	Induces T cell responses that protect mice from IAV challenge	Preclinical	(36, 37)
	Peptide-loaded virosomes	Minimal T cell epitope from M1	Virosome, CpG-ODN 1826	Induces peptide-specific CD8^+^ T cells that reduce body weight loss of mice after heterologous IAV infection	Preclinical	(38)
	FP-01.1	Long peptides containing T cell epitopes from M1, NP, PB1, and PB2	Peptides conjugated to fluorocarbon moiety	Induces CD4^+^ and CD8^+^ T cells in healthy adults that are cross-reactive against IAV-infected target cells	Phase I trials	(39)
	Flu-v	Long peptides containing T cell epitopes from M1, M2, and NP	Montanide ISA 51VG	Induces peptide-specific CD8^+^ T cells in healthy adults	Phase I trials	(40)

Virus-like particle/viral vector vaccine	Peptide fused to PapMV nanoparticles	T cell epitope from NP	Papaya mosaic virus nanoparticles	Induces peptide-specific CD8^+^ T cells in mice	Preclinical	(41)
	DdFluM1	T cell epitopes from M1	Adenoviral dodecahendron particles	Induces peptide-specific CD4^+^ and CD8^+^ T cells in chickens	Preclinical	(42)
	PIV5-NP	T cell epitope from NP	Parainfluenza 5	Induces CD8^+^ T cells in mice that reduce morbidity and lethality after IAV challenge	Preclinical	(43)
	MVA-NP + M1	T cell epitopes from M1 and NP	Modified vaccinia virus Ankara vector	Induces influenza-specific cellular responses in healthy adults and elderly that reduce viral shedding and reduction of symptoms	Phase II trials	(44–46)

DNA vaccine	DNA plasmids encoding for T cell epitopes	DNA encoding for B and T cell epitopes from HA and NP	None	Induces T cell responses that reduce body weight loss of mice after IAV challenge	Preclinical	(47)

### Live attenuated influenza vaccines

Live attenuated influenza vaccines (LAIV) are currently on the market as intranasal (i.n.) IAV vaccines. LAIV induce next to humoral responses both CD4^+^ and CD8^+^ T cells in children (26, 27). Remarkably, no cellular immune responses are detected in adults receiving LAIV; the cause of this discrepancy might be related to the naïve status of children. Furthermore, LAIV are more effective than current seasonal trivalent inactivated influenza vaccines (TIV) in children but not in adults, suggesting that the induction of cellular immune responses increases the efficacy of LAIV (48). The encapsulation of LAIV in a biopolymer of alginate and subsequent subcutaneous (s.c.) administration-induced CD8^+^ T cell responses that protected mice from a heterologous IAV challenge (49), indicating that LAIV can induce T cell responses via immunization routes other than i.n. by use of formulation strategies. The induction of cellular responses by LAIV might be explained by the “live” state of the vaccine antigen; it can still infect after vaccination. During the viral replication, many viral proteins containing CD8^+^ and CD4^+^ epitopes are produced within the infected host cell, leading to efficient antigen processing via the endogenous pathway, which leads to MHC class I presentation and subsequent T cell activation.

### Whole inactivated influenza virus

Like LAIV, whole inactivated influenza virus (WIV) contains internal proteins such as nucleoprotein (NP), matrix proteins 1 and 2 (M1 and M2, respectively), polymerase basic proteins 1 and 2 (PB1 and PB2, respectively) and polymerase acidic protein (PA), which possess conserved T cell epitopes. WIV vaccines were replaced by subunit and split vaccines due to incidence of adverse events associated with WIV (50), but have been given increased attention the past few years in the search for cross-reactive vaccines (51). Improvements on WIV production and purification methods have decreased WIV-associated side effects, making this vaccine acceptable for use again, especially for the induction of broadly reactive immune responses. At normal clinical dose, which typically does not exceed 15 μg of HA protein, WIV induces adequate neutralizing antibody titers, but generally fail to induce any cellular responses regardless of administration route (52). However, studies by Budimir et al. showed that multiple high doses of WIV, such as two times 6 μg, were able to induce significant amounts of IAV-specific CTLs in mice (53–55). The critical roles of membrane fusion activity and the presence of viral ssRNA for the induction of CTLs were established (53, 55). Intramuscular (i.m.) administration of WIV proved to be more effective at inducing CTLs than i.n. administration (54). This was confirmed by Takada et al., who found that intranasal vaccination with WIV failed to induce T cell responses (56). By contrast, one study utilizing gamma-irradiated WIV showed that the protective effect of WIV was mainly mediated by T cell responses (29). It is suspected that the method of WIV inactivation can have an effect on its immunogenicity. Aside from increased dosage, WIV-induced cellular responses can also be boosted by the addition of adjuvants. For instance, a dose of 2.5 μg WIV adjuvanted with cationic lipid/DNA complex (CLDC) was able to induce influenza-specific CD4^+^ and CD8^+^ T cell responses in mice, whereas alum adjuvanted WIV only induced high-antibody responses (57). Similar to studies with WIV, the addition of alum to virosomes proved to be detrimental to cellular responses in mice (58), since it skewed the T_H_ to a T_H_2-type response.

### Virosomes

Virosomal vaccines can also induce influenza-specific CTL responses. The addition of adjuvants to virosomes is necessary to induce T cell responses, since unadjuvanted virosomes only induce humoral responses. The incorporation of LpxL1, a detoxified lipopolysaccharide, in virosomes significantly increased IFN-γ secretion in mice (59). Madhun et al. showed that addition of the saponin-based Matrix-M adjuvant to virosomes significantly increased the production of T_H_1-associated cytokines IL-2 and IFN-γ when administered i.m. to mice (60). Strikingly, a significant induction of multifunctional CD4^+^ T cells was also observed in a murine model after the addition of Matrix-M to the virosomal vaccine. In a similar study, Radosevic et al. screened multiple adjuvants (i.e., aluminum phosphate, aluminum hydroxide, MF59, and Matrix-M) in combination with virosomes in mice (61). Unlike the study by Madhun et al., virosomes were readily able to induce CD4^+^ T cells, and addition of any adjuvant, including Matrix-M, did not increase these responses. However, only MF59 and Matrix-M adjuvanted virosomal vaccines were able to induce IAV-specific CD8^+^ T cell responses. Furthermore, addition of any aluminum salt-based adjuvants proved to be ineffective at eliciting any cellular responses, which was probably due to T_H_2-skewed immune responses by aluminum salts.

The ability to induce cellular immune responses by some marketed influenza vaccines is of great value in order to offer limited cross-reactivity against non-matched influenza strains. These vaccine formulations can play a role as an intermediate solution until the next generation of cross-protective influenza vaccines is developed.

### Peptide antigens

Peptides are another type of antigen that can be used in T cell-inducing influenza vaccines. However, short peptides that consist of a minimal epitope are generally not immunogenic, and thus require additional modification or formulation to be able to induce T cell responses (62).

Several preclinical studies have used minimal epitope peptides as their main antigen to induce influenza-specific cellular responses. Short influenza peptides conjugated to phosphatidylserine were able to induce CD8^+^ T cell responses in mice (35). The conjugation of lipids to peptides opens up several possibilities; a PA-derived peptide conjugated to Pam2Cys, a lipid and TLR2 ligand, and efficiently induced peptide-specific CTL responses in mice (63). Furthermore, peptides conjugated to liposomes were able to minimize morbidity in IAV-infected mice through the induction of CD8^+^ T cells (36, 37). Remarkably, these peptide–liposome conjugates were able to induce CD8^+^ memory T cells without the contribution of CD4^+^ T cells. Liposomes act as a delivery system for the peptides, which are then internalized more efficiently by APCs than unformulated peptides. Direct conjugation of the peptide to a lipid or liposome is, however, not required. NP_366–374_ peptide encapsulated in liposomes was able to induce potent T cell responses when mixed with anti-CD40 mAbs, and reduced viral lung titers of influenza-infected mice (64).

Aside from liposomes, virosomes have also been used as delivery systems for short peptide antigens. These virosomes utilize the membrane fusion activity of HA proteins to deliver the loaded peptide to the cytosolic compartment of the APC. An early study showed that virosomes loaded with the H-2K^d^ binding influenza NP_147–155_ peptide-induced CTLs that were able to lyse IAV-infected target cells (65). The addition of the adjuvant CpG-ODN 1826 to influenza M1_58–66_ peptide-loaded virosomes was shown to increase peptide-specific CD8^+^ T cell responses even further (38), which resulted in a faster recovery of vaccinated mice after heterologous influenza virus infection.

Long peptide vaccines consisting of multiple epitopes are, opposed to short peptide vaccines, already in the clinical testing phase. Flu-v consists of an equimolar mixture of four synthetic polypeptides derived from M1, M2, and NP IAV proteins, formulated with the adjuvant Montanide (40). Flu-v-induced peptide-specific T cells in healthy subjects; unfortunately, reactivity against actual IAV strains was not determined. However, vaccination studies in mice showed that CD8^+^ T cell responses induced by Flu-v did reduce mortality after IAV infection (66).

Similar to Flu-v, FP-01.1 consists of six polypeptides derived from M1, NP, PB1, and PB2, which were conjugated to a fluorocarbon moiety. The vaccine was able to induce CD4^+^ and CD8^+^ T cells in healthy subjects (39). Moreover, these T cells were cross-reactive with H1N1 and H3N2 IAV-infected target cells. This is the first study that shows a peptide vaccine capable of inducing cross-reactive T cells in humans, which is very encouraging for the development of cross-reactive T cell-inducing vaccines.

The studies described above suggest that peptide-based approaches are very promising in the development of T cell-inducing IAV vaccines. However, an important challenge is the genetic variability among the human population in relation to epitope recognition and presentation. CD4^+^ and CD8^+^ T cells recognize IAV epitopes displayed on MHC molecules, which are called human leukocyte antigen (HLA) molecules in humans. Different HLA polymorphisms occur in the human genome, resulting in a host of varying HLA molecules in the human population. Each HLA can only bind specific viral epitopes, which means that multiple epitopes of the same antigen need to be in a peptide-based vaccine to cover the human population (67). *In silico* prediction methods can be employed to determine the potential T cell immunogenicity of conserved epitopes across multiple IAV strains (68). Furthermore, several transgenic mouse strains have been bred that express HLA molecules, which can be used in preclinical development. Nonetheless, there remains a significant challenge for peptide-based vaccines to include enough epitopes to cover each HLA type, which would be required for a vaccine to be effective in the entire population.

### Other T cell influenza vaccine concepts

Aside from the vaccine strategies described above, several other concepts are currently in clinical development (Table 1). Multimeric-001 is a synthetic recombinant protein composed of nine T cell and B cell epitopes derived from HA, NP, and M1 influenza proteins (31). The vaccine in combination with the adjuvant Montanide ISA 51VG was able to induce cellular responses in healthy subjects. The cellular responses showed limited reactivity to multiple IAV strains. In a follow-up study, the Multimeric-001 vaccine showed an induction of humoral and cellular responses in elderly subjects similar to responses observed in healthy adults (32). While the results of these studies are encouraging, the true effectiveness of the induced cellular responses against homologous and heterologous IAV infections has yet to be determined.

Another concept, which has advanced to the clinical stage of development, is the modified vaccinia virus Ankara vectored vaccine MVA-NP + M1 (45). This vaccine consists of a vaccinia virus Ankara expressing the influenza proteins NP and M1. Several clinical trials, including a phase II study, were conducted with this vectored vaccine. MVA-NP + M1 was able to expand pre-existing memory CD8^+^ T cells in both healthy adults and elderly, and also increased the IAV-specific CD4^+^ T cell population (44, 46).

### T cell-based influenza vaccine concepts in the clinical phase

The protein-based influenza vaccines such as LAIV, WIV, and virosomes currently have the advantage that they are already licensed and have been widely used. Such vaccines might be excellent candidates to prime naïve populations for both cellular and humoral responses.

Peptide-based vaccine concepts have the advantage that they can be easily engineered and produced synthetically. However, as mentioned above, selection of the right epitopes remains vital. These vaccines also require additional formulation with adjuvants to increase their immunogenicity. Nonetheless, several peptide-based vaccines have entered the clinical phase.

Vectored T cell-inducing vaccines are a sophisticated concept. They include both antigen and adjuvant in a single particle. Since they express whole proteins rather than epitopes, vectored vaccines might have a higher coverage among different populations compared to peptide-based vaccines. A recent study also combined a seasonal influenza vaccine with MVA-NP + M1 to increase the breadth of the immune response (69). Such an approach is a major improvement and might be an ideal solution to induce both humoral and cellular immunity with a single vaccine. Other concepts, such as peptide-based influenza vaccines, are also eligible to be used simultaneously with seasonal influenza vaccines, as demonstrated recently (34). This is a good step toward a universal influenza vaccine.

## Vaccine Priming

The IAV-naïve status and age of persons may influence the immunogenicity of T cell-inducing IAV vaccines. This was already observed with LAIV vaccines, which effectively induce cellular responses in naïve children, but not induce such responses in adults, who already established an immunological memory to IAV (26, 27). A study in mice reported that CD8^+^ T cells primed by LAIV rapidly differentiated to IAV-specific memory T cells after short-interval boosting, and were able to protect against heterologous challenge (70). Several T cell-inducing vaccine concepts consider the potency of the prime-boost approach; a DNA–protein prime-boost concept enhanced the T cell responses to IAV in mice (71), and in a clinical trial priming with Multimeric-001 before a seasonal influenza vaccine boost greatly increased IAV-specific cellular responses in elderly subjects (32). Priming at an early age in naïve mice with IAV resulted in the induction of long-term memory CD8^+^ T cells with the broadest reactivity, while priming at an older age resulted in a CD8^+^ T cell population with a reduced diversity (72). Thus, T cell priming at an early age, when the subject is still naïve, should be considered before immunization with an influenza vaccine that only induces humoral responses. As a result, the intended target population of a vaccine is key for vaccine design and development (73).

## Resident Lung T Cells

Many T cell-inducing vaccine concepts aim for the induction of systemic IAV-specific T cell responses. However, local T cell responses at the site of IAV infection are potentially more effective. The presence of IAV-specific resident memory T cells (T_RM_) in the lungs was correlated with clearance of heterologous IAV infection in mice (74). CD4^+^ T cells mediated the formation of CD8^+^ T_RM_ cells, adding yet another important function for CD4^+^ T_H_ (75). Current knowledge on the establishment of T_RM_ cells has been reviewed recently (76). While the process of T_RM_ induction is not completely unraveled, some possible mechanisms can be exploited to induce IAV-specific T_RM_ responses with vaccines. A recent study specifically targeted an antigen to resident lung DCs using antibodies, and were able to generate IAV-specific CD8^+^ T_RM_ cells in mice that provided protection against a lethal influenza challenge (77). Furthermore, it is known that CXCR3-expressing CD8^+^ T cells play an important role in the establishment of CD8^+^ T_RM_ cells in the lungs (78). The near future may learn us whether specific targeting of certain T cell populations, e.g., by adjusting the route of administration to the lungs (79, 80), may add to the potential of T cell-inducing influenza vaccines.

## Preclinical Cellular Correlates of Protection

There is clear evidence that cellular responses correlate with a reduction of symptoms after IAV infection. However, current correlates of protection (CoP) for influenza vaccines are all based on the induction of antibodies, such as the presence of hemagglutination inhibition- or virus neutralization titers, which are inadequate CoPs for T cell-inducing vaccines. Instead, responses that indicate the presence of effector T cells such as IFN-γ and IL-10 cytokines, combined with cytotoxic effector molecules like granzyme B may be more suitable as CoP for T cell-inducing vaccines (81). These parameters also need to be further evaluated in epidemiological studies in order to define their efficacy. For instance, it is still unclear what quantitative levels of IAV-specific CD8^+^ or CD4^+^ T cell responses are required for protection against an IAV challenge. Furthermore, an adequate translation from animal models to the human setting has to be made. While there is quite some experience with humoral responses against IAV in animal models and their relation to the clinic, such experience has not been established yet for cellular responses. Establishing these responses as human CoPs, and translating study findings from animal models to humans remain important tasks for the development of T cell-inducing IAV vaccines.

## Concerns and Limitations of T Cell-Inducing IAV Vaccines

There are some concerns whether IAV-specific T cells can provide the same level of protection compared to IAV-specific antibodies. While T cells have a broader reactivity, they can only recognize and lyse IAV-infected host cells. Most likely, an IAV infection is already spreading before an efficient T cell response is mounted. It can therefore be debated whether T cells responses actually provide protection (i.e., sterilizing immunity) or only shorten the length and severity of influenza symptoms (i.e., decreased morbidity). The difference between these two can be very hard to distinguish. Therefore, elucidation of T cell responses after influenza infection in humans is of critical importance to determine the efficacy of T cell-inducing influenza vaccines. Nonetheless, reduction of morbidity of IAV infections would already be a great success in situations where seasonal influenza vaccines would be ineffective, such as a mismatched influenza epidemic or an influenza pandemic. The definition of protection should therefore not only be limited to sterilizing immunity but also to reduction of disease morbidity.

Another concern is the possibility of excessive T cell responses to IAV infections, which could cause immunopathology in the lungs (82). There are indications that excessive T cell responses mediate severe lung inflammation and subsequent lung damage after IAV infection in mice. Only one study describes the phenomenon in humans; elevated IAV-specific CD8^+^ and CD4^+^ T cell responses were found in pandemic 2009 H1N1-infected children with severe pneumonia (83). It was, however, unclear whether these T cell responses were the cause of pneumonia or simply present due to the infection.

It is yet unknown whether T cell-inducing influenza vaccines can mount long-lasting T cell responses after a limited number of immunizations. As already discussed above, natural IAV infections are able to induce T cell responses, but their effectivity is limited. Studies suggest that local inflammation and inflammatory cytokine production caused by IAV infection suppress CD8^+^ T cell responses in mice. This was partly attributed to an increased expression of PD-L1 on the CD8^+^ T cells, which cripples the functionality of these T cells (84, 85). T cell-based vaccines, however, should not experience the effects of these immunosuppressive pathways, since inflammation after immunization is generally limited. It is thus likely that these vaccines can induce T cell responses, which are more potent than those elicited by natural IAV infections. Nonetheless, it is important that T cell-inducing vaccines elicit balanced T cell responses, and special interest should be given to T cell-mediated immunopathology during safety studies of these vaccines.

Aside from the intensity of T cell responses, special attention should be given to the selection of target epitopes derived from IAV. A recent study described the existence of tolerizing epitopes in certain influenza strains, which are recognized by autologous regulatory T cells and may suppress protective T cell responses (86). Another study found that T cells against certain immunodominant epitopes such as M1_58–66_ have a poor functionality, and are unable to clear IAV-infected cells (87). It was hypothesized that these immunodominant epitopes are actually a decoy of IAV to evade T cell-mediated immunity and to prevent the generation of more potent T cells against other epitopes. It is therefore important that such epitopes, which could lead to decreased or impotent T cell responses, are identified and excluded in any prospective T cell-inducing IAV vaccines.

## Conclusion

Humoral immune responses elicited by current IAV vaccines do not provide sufficient cross-protection against non-matched IAV infections. IAV-specific T cells recognize conserved epitopes of IAV and thus have to potential to be cross-protective. Many different T cell-inducing vaccines are currently under development, and some have even reached clinical phases. Selecting suitable preclinical testing models and clinical CoPs are vital for further development of such vaccines. In addition, proper understanding the effectiveness of each T cell response and their possible pathological effects is of great importance. The current developments with T cell-inducing IAV vaccines, including novel formulations and extended immunological insight, are fast evolving and may ultimately result in universal influenza vaccines.

## Conflict of Interest Statement

The authors declare that the research was conducted in the absence of any commercial or financial relationships that could be construed as a potential conflict of interest.
